# Supplementary material for: Prediction uncertainty assessment of a systems biology model requires a sample of the full probability distribution of its parameters
Source: PeerJ. 2014 Jun 17;2:e433. doi: 10.7717/peerj.433 (PMC4081139; doi:10.7717/peerj.433)
Supplement: Supplemental Information 3 — This pdf contains further detailed information on the main article. [file peerj-02-433-s003.pdf]

Supplemental Information to: Prediction uncertainty assessment of a systems biology model requires a sample of the full probability distribution of its parameters

Simon van Mourik[1], Cajo ter Braak[2], Hans Stigter[3], and Jaap Molenaar[4]

[1,2,3,4]**Plant Sciences Group, Wageningen University and Research Center, Wageningen, The Netherlands**
[1,4]**Netherlands Consortium for Systems Biology, Amsterdam, The Netherlands**

ABSTRACT

Keywords:

General settings

The models are calibrated on a time series of 10 equidistantly sampled data points generated. The MCMC algorithm was carried out in log space with a log-uniform prior distribution for the parameters with a cutoff (Grandison and Morris, 2008), in particular: $p(\log(\theta)) = 1$ for $\theta \in [10^{-6}\theta^0, 10^6\theta^0]$, and $p(\log(\theta)) = 0$ elsewhere, with θ^0 an initial guess of the parameter value. For the illustrative example we used a uniform prior for better illustration of the confidence region. For the likelihood, we assumed Gaussian noise with $\sigma = 0.1y_d$. The data was generated without noise in the simulations (Gutenkunst et al., 2007b), with θ^{PML} equal to the true parameter values and also θ^0 was set to the true value.

Time integrations were carried out in the Matlab environment using the ode15s command. DE-MCz was carried out with 4 chains, a thinning rate $K = 10$, and in total $4\ 10^5$ iterations of which $1\ 10^5$ were used for burn-in. We used Gelman's $\hat{R}$ statistic to check for convergence (Gelman and Rubin, 1992).

The Q distribution was computed using 1000 samples from $\pi(\theta)$. Q was computed in (7) using Riemann summation with 100 time points. Approximating the integral with only 30 points had practically no effect on the outcomes. When time integrations failed to converge, the Q_{95} value was set to zero and not displayed.

Extremely low or high values of y can lead to extreme differences, which tend to dominate Q, sometimes even if $y_p(\theta)$ and $y_p(\theta^{PML})$ render the same biological implication. To prevent this, we considered only y values within a range $[y^{min}(t), y^{max}(t)]$ in which differences are assumed to be still biologically relevant. We used $y(t)^{min} = 10^{-6}$ and $y(t)^{max} = 10^6$.

Linearized covariance analysis

LCA is based on a quadratic approximation of the log posterior using first order sensitivities of the predicted output towards parameter changes. For any time point t, the

standard deviation on the maximum likelihood prediction $y(t,\theta^{ML})$ is estimated by (Gutenkunst et al., 2007a)

$$\sigma^2(y(t,\theta^{ML})) = \sum_{i,j} \frac{\partial y(t)}{\partial \theta_i}(H^{-1})_{i,j}\frac{\partial y(t)}{\partial \theta_j}\Big|_{\theta^{ML}}. \quad (1)$$

Here $H_{i,j}(\theta^{ML}) = \frac{d^2\chi^2(\theta^{ML})}{d\theta_i d\theta_j}$ is the Hessian. This assumes a symmetric distribution of $y(t)$ around the maximum likelihood prediction. Assuming a normal distribution, the 95% confidence intervals of $y(t)$ are then $y(t,\theta^{ML}) \pm 1.96\sigma(y(t,\theta^{ML}))$. Replacing the linear derivatives in (1) with logarithmic derivatives gave similar results in Fig. 2D.

The models from the BioModels database, and the data sets

The following models were taken from the BioModels database (Li et al., 2010): BIOMD0000000229, -011, -003, -005, -035, and -021. Model 1 describes a protein network that produces spontaneous oscillations in excitable cells of Dictyostelium (Laub and Loomis, 1998). It has 7 variables and 14 parameters. We took $T = 15$, and $x(0) = [3.39, 2.45, 1.6, 1.2, 1.13, 0.9, 0.48]$. Model 2 describes a signaling pathway, modeled as a basic 3- stage Mitogen Activated Protein Kinase (MAPK) cascade in solution (Levchenko et al., 2000). It has 22 variables and 30 parameters. We took $T = 100$ and $x(0)$ =[0.4,0,0,0.3,0,0,0,0,0.2,0, 0.2,0,0,0,0,0,0.3,0.2,0,0.3,0,0]. Model 3 describes a minimal cascade model for the mitotic oscillator involving cyclin and cdc2 kinase (Goldbeter, 1991). It has 3 variables and 10 parameters. We took $T = 25$ and $x(0) = [0.01, 0.01, 0.01]$. Model 4 describes a model of the interactions of cdc2 and cyclin (Tyson, 1991). It has 6 variables and 8 parameters. We took $T = 50$ and $x(0) = [0, 0.75, 0, 0.25, 0, 0]$. Model 5 describes a genetic circadian oscillator model (Vilar et al., 2002). It has 9 variables and 16 parameters. We took $T = 50$ and $x(0) = [0, 0, 1, 0, 1, 0, 0, 0, 0]$. Model 6 describes circadian oscillations of the PER and TIM proteins in *Drosophila* (Leloup and Goldbeter, 1999). It has 10 variables and 44 parameters. We took $T = 50$ and $x(0)$ =[0.0341,0.0341,0.0304,0.0304, 0.0257,0.0257,0.2091,1.1551,0.1483,0.1483]. For models with oscillating dynamics we created data sets with a time span that covered less than two oscillations for all variables, to avoid loss of information on fast dynamics due to a fixed amount of time points. For multi-variable models, the time series of individual variables were concatenated. The data vector so obtained was used in the likelihood calculations.

The influence of the size of prediction perturbations and base b on the range of $Q_{0.95}$

The range sizes in which the prediction uncertainties lie, is quite robust towards changing the conditions under which the predictions were generated. For each prediction a parameter is multiplied with a factor 100 or 0.01. We varied this factor. Repeating the simulation experiment (Fig. 3A) with factor 1000 and 0.001, and with factor 10 and 0.1, gave similar results. The sizes of the intervals, and the maximum prediction uncertainty for each model remained mostly of the same order of magnitude (Fig. S1).

The choice of base b reflects which differences in a prediction are considered relevant and thus influences the magnitude of prediction uncertainty, but it does not change the range of uncertainties on a logarithmic scale as in Fig. S1. In this study we used $b = 2$ in

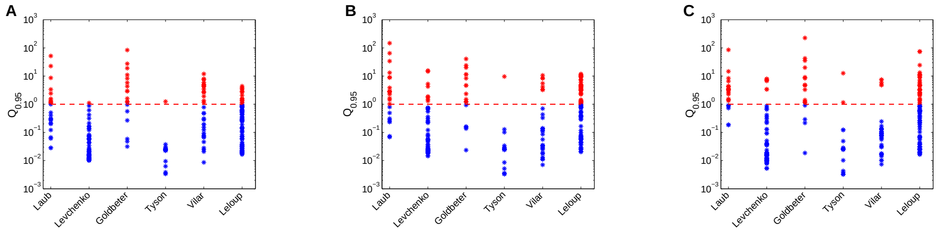

Figure 1. Influence of varying the perturbation factor. A) factor 10. B) factor 100. C) factor 1000.

equation (7), allowing only relative errors of order 2 or higher to appreciably contribute to Q. Increasing the base from b to c ($c > b$) decreases Q - and therefore Q_α - with a factor $(\log_b(c))^2$, but does not change the relative differences between the Q_α values. Hence, the intervals shift downward. For example, increasing the base from 2 to 5 decreases Q_α with a factor 5.4.

Model order reduction

The computational effort needed to sample $\pi(\theta)$ may be large due to the model integrations required to compute the likelihood $\chi^2(\theta)$. A reduction method that is commonly applied (Gutenkunst et al., 2007b,a; Brown and Sethna, 2003; Brown et al., 2004) is to circumvent the integration step by locally approximating the χ^2 function with a second order Taylor expansion. In this way, time integrations only have to be carried out to compute the Hessian. The reduction works as follows. The maximum likelihood parameter vector minimizes $\chi^2(\theta)$, so $\frac{d\chi^2}{d\theta}(\theta^{ML}) = 0$, and the second order Taylor expansion around θ^{ML} reads

$$\chi^2(\theta) \approx \chi^2(\theta^{ML}) + \frac{1}{2}\Delta \log \theta^T H(\theta^{ML}) \Delta \log \theta, \tag{2}$$

where $\Delta \log \theta = \log(\theta) - \log(\theta^{ML})$ and $H_{i,j}(\theta^{ML}) = \frac{d^2\chi^2(\theta^{ML})}{d\log(\theta_i) d\log(\theta_j)}$, the positive semidefinite Hessian. The identity $H = 2J^T J$, with $J_{i,j} = \frac{dy_i}{\sigma_i d\log(\theta_j})$ simplifies numerical computations (Brown and Sethna, 2003). It is recommended to use a logarithmic derivative (Gutenkunst et al., 2007b), since parameter values may vary over orders of magnitude. The parameters are approximately distributed as

$$\log(\theta) \sim N(\log(\theta^{ML}), 2H(\theta^{ML})^{-1}), \tag{3}$$

and a sample can be drawn directly from this distribution, i.e., without the need for MCMC sampling. We compared the prediction uncertainties estimated with and without the reduction (Fig. S2). As could be expected, the $Q_{0.95}$ ranges are affected by the approximation errors. Especially for the Leloup model, the model order reduction induces large errors in the estimated prediction uncertainty. In practice, errors in estimating θ^{ML} may further affect the estimated prediction uncertainty.

Computational costs

We used three different methods: full MCMC, model order reduction, and linearized covariance analysis (LCA). MCMC sampling time depends on the number of iterations,

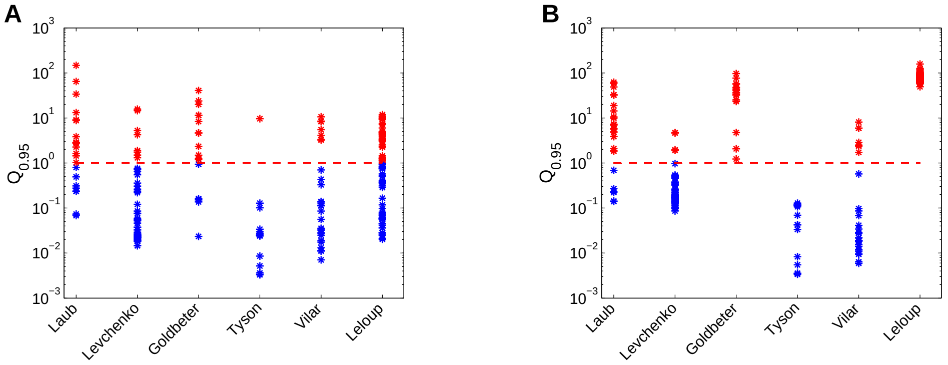

Figure 2. A) $Q_{0.95}$ values obtained via model integrations, and B) via model order reduction.

and the time needed for drawing from a normal distribution depends largely on checking whether a draw is in or outside the region defined by the prior, which in turns depends on the size of the sample. The most time LCA and the model order reduction method need, is for the p time integrations to compute the Hessian, with p the number of parameters. This is much smaller than the number of iterations needed (here a factor of about 10^4.

Robustness of prediction uncertainty with respect to size of the posterior sample

Throughout this paper, $Q_{0.95}$ is computed using 1000 samples representing $\pi(\theta)$. To check whether this does not influence the qualitative outcomes, the $Q_{0.95}$ values are compared with those obtained via 400 samples (Fig. S3), using the approximation in (2) to reduce computational costs. The sizes and locations of the intervals hardly differ with the sample size.

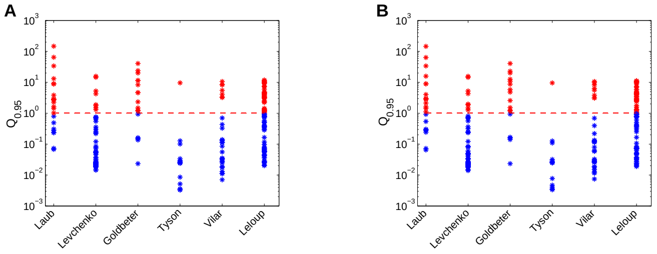

Figure 3. A) $Q_{0.95}$ values obtained with 1000 samples from $\pi(\theta)$. B) $Q_{0.95}$ values obtained with 400 samples.

REFERENCES

Brown, K. S., Hill, C. C., Calero, G. A., Myers, C. R., Lee, K. H., Sethna, J. P., and Cerione, R. A. (2004). The statistical mechanics of complex signaling networks: nerve growth factor signaling. *Physical biology*, 1(3):184.

Brown, K. S. and Sethna, J. P. (2003). Statistical mechanical approaches to models with many poorly known parameters. *Physical Review E*, 68(2):021904.

Gelman, A. and Rubin, D. B. (1992). Inference from iterative simulation using multiple sequences. *Statistical science*, pages 457–472.

Goldbeter, A. (1991). A minimal cascade model for the mitotic oscillator involving cyclin and cdc2 kinase. *Proceedings of the National Academy of Sciences*, 88(20):9107–9111.

Grandison, S. and Morris, R. J. (2008). Biological pathway kinetic rate constants are scale-invariant. *Bioinformatics*, 24(6):741–743.

Gutenkunst, R. N., Casey, F. P., Waterfall, J. J., Myers, C. R., and Sethna, J. P. (2007a). Extracting falsifiable predictions from sloppy models. *Annals of the New York Academy of Sciences*, 1115(1):203–211.

Gutenkunst, R. N., Waterfall, J. J., Casey, F. P., Brown, K. S., Myers, C. R., and Sethna, J. P. (2007b). Universally sloppy parameter sensitivities in systems biology models. *PLoS computational biology*, 3(10):e189.

Laub, M. T. and Loomis, W. F. (1998). A molecular network that produces spontaneous oscillations in excitable cells of Dictyostelium. *Molecular biology of the cell*, 9(12):3521–3532.

Leloup, J. C. and Goldbeter, A. (1999). Chaos and birhythmicity in a model for circadian oscillations of the PER and TIM proteins in Drosophila. *Journal of Theoretical Biology*, 198(3):445–459.

Levchenko, A., Bruck, J., and Sternberg, P. W. (2000). Scaffold proteins may biphasically affect the levels of mitogen-activated protein kinase signaling and reduce its threshold properties. *Proceedings of the National Academy of Sciences*, 97(11):5818–5823.

Li, C., Donizelli, M., Rodriguez, N., Dharuri, H., Endler, L., Chelliah, V., Li, L., He, E., Henry, A., Stefan, M. I., et al. (2010). Biomodels database: An enhanced, curated and annotated resource for published quantitative kinetic models. *BMC systems biology*, 4(1):92.

Tyson, J. J. (1991). Modeling the cell division cycle: cdc2 and cyclin interactions. *Proceedings of the National Academy of Sciences*, 88(16):7328–7332.

Vilar, J. M., Kueh, H. Y., Barkai, N., and Leibler, S. (2002). Mechanisms of noise-resistance in genetic oscillators. *Proceedings of the National Academy of Sciences*, 99(9):5988–5992.
